# Bio-Catalysis in Multicomponent Reactions

**DOI:** 10.3390/molecules25245935

**Published:** 2020-12-15

**Authors:** Ndze Denis Jumbam, Wayiza Masamba

**Affiliations:** Department of Chemical and Physical Sciences, Faculty of Natural Sciences, Walter Sisulu University, Nelson Mandela Drive, Mthatha 5117, South Africa; njumbam@wsu.ac.za

**Keywords:** bio-catalysis, diversity-oriented chemistry, enzyme, multicomponent reactions, whole-cell catalyst

## Abstract

Enzyme catalysis is a very active research area in organic chemistry, because biocatalysts are compatible with and can be adjusted to many reaction conditions, as well as substrates. Their integration in multicomponent reactions (MCRs) allows for simple protocols to be implemented in the diversity-oriented synthesis of complex molecules in chemo-, regio-, stereoselective or even specific modes without the need for the protection/deprotection of functional groups. The application of bio-catalysis in MCRs is therefore a welcome and logical development and is emerging as a unique tool in drug development and discovery, as well as in combinatorial chemistry and related areas of research.

## 1. Introduction

Recent advances in organic synthesis, fueled by the need for more efficient, environmentally compatible processes, have led to the development of new strategies. Multicomponent reactions (MCRs) are one such strategy. In these reactions, three or more components react together in one pot, generating a rapid assembly of high-complexity molecular architectures resulting in a single-molecular entity containing most, if not all, the starting materials components. Compared to standard organic synthesis wherein compounds are prepared individually and sequentially, consisting of isolation/purification steps that oftentimes generate waste products, MCRs allow for a fast construction of complex and diverse molecular structures in a convergent manner under mild conditions in a single step, leading, in many instances, to the formation of heterocyclic compounds [1,2,3,4]. A unique characteristic of MCRs is their simplicity and atom efficiency, high selectivity and low toxicity of readily available starting materials. Therefore, the general principles of green chemistry can easily be applied to MCRs [5]. 

Given the above characteristics, MCRs have experienced an exponential growth in recent years due to their numerous synthetic applications in pharmaceuticals in fine chemicals, as well as in drug discovery and optimization [6,7]. 

Multicomponent reactions have been known since a long time. The Strecker reaction is the first documented MCR whereby α-amino acids were prepared from an aldehyde or ketone, ammonia and hydrogen cyanide [8,9]. Since then, many other MCRs have been discovered, and there is a multitude of these reactions today, which include, among others, the Asinger reaction, Biginelli reaction, the Hantzsch pyridine synthesis, Mannich, Strecker and Ugi [10,11].

Most MCRs are mediated by a variety of catalysts [12,13,14]. Although many of them are important industrial organic reactions, the development of biocatalytic MCRs has only recently attracted the interest of organic chemists [15]. Few direct bio-catalyzed asymmetric MCRs have been reported in the literature. 

Many enzymes exhibit diverse catalytic activity that goes beyond their natural substrates. This phenomenon, known as enzyme promiscuity [16], can be easily exploited and applied in MCRs. Enzymes are ecofriendly, biodegradable and their reactions occur under mild conditions [17], thus outperforming most traditional chemical catalysts. When immobilized on solid supports [18], they can be recovered and reused in multiple transformations. Enzymes, therefore, are becoming a better alternative to chemical catalysts and are ideal in MCRs.

## 2. The Asinger Reaction

**Scheme 1:**
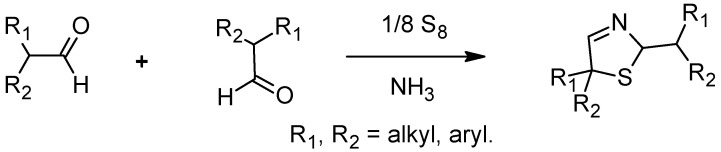
Asinger reaction.

The Asinger reaction involves the synthesis of 3-thiazoline derivatives originally from two molecules of a ketone, one molecule of sulfur and one molecule of gaseous ammonia [19,20]; today, α-haloaldehydes or α-haloketones, ammonia, NaSH and aldehydes or ketones are usually the starting materials in the reaction [21,22,23], allowing for the simultaneous formation of the 3-thiazoline ring structure (Scheme 1). 

**Scheme 2:**
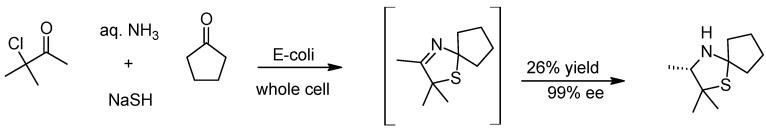
Asinger synthesis of (*S*)-2,2,3-trimethyl-1thia-4-azaspiro[4.4]nonane.

Though discovered in 1956, its full synthetic value was recognized only recently thanks to the advances made in MCRs. The 3-thiazolidine ring is an interesting scaffold contained in many bioactive molecules [24,25] and serves as an industrial key intermediate for the production of d-penicillamine [26] and represents a structural motif in a range of HIV protease inhibitors [27]. Zumbrägel and Gröger developed the first and only bio-catalyzed enantioselective synthesis of this ring system [28] and prepared (*S*)-2,2,3-trimethyl-1-thia-4-azaspiro[4.4]nonane via a one-pot process, leading to moderate conversion and excellent enantioselectivities (99% ee) using *Escherichia coli* whole cells as the catalyst. The reaction proceeded via a 3-thiazolidine ring intermediate (Scheme 2), which was subsequently bio-catalytically reduced by an imine reductase in combination with a glucose dehydrogenase and glucose for in situ cofactor recycling.

An overall moderate conversion of 26% is due to the limited diffusion of the thiazolidine in the Asinger reaction. This limitation not withstanding represents an improvement compared to the compartmentalized one-pot process that afforded only 13% yield.

## 3. The Biginelli Reaction

**Scheme 3:**
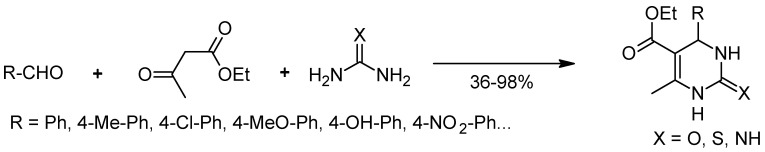
Preparation of different dihydropyrimidine derivatives via the general Biginelli reaction.

The Biginelli reaction was initially used to allow an efficient access to multi-functionalized 3,4-dihydropyrimidin-2(1*H*)-ones (DHPMs) [29] from aldehyde-urea derivatives of aceto- and oxaloacetic acids. The relatively harsh acidic conditions involved in the reaction led the search for greener enzyme-catalyzed versions. The reaction quickly became one of the major MCRs as a route to many heterocyclic compounds, including 4-dihydropyrimidin-2(1*H*)-thiones, as well as intermediates in the Hantzsch pyridine synthesis [30,31] (Scheme 3).

**Scheme 4:**
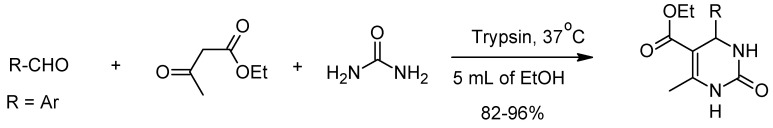
Preparation of different dihydropyrimidinones via the enzyme-catalyzed Biginelli reaction.

Since both urea and the β-ketoester are bifunctional groups, an MCR would lead to the formation of five new bonds in the end products and constitutes an efficient method to access these derivatives. A biocatalytic Biginelli three-component reaction was developed for the high-yield synthesis of 3,4-dihydropyrimidine-2-(1*H*)-ones, consisting of the condensation of urea or thiourea with a substituted benzaldehyde and a 1,3-ketoester in aqueous phosphate buffer and using *Saccharomyces cerevisiae* as a biocatalyst [15]. Baker’s yeast was later found to successfully catalyze the one-pot synthesis of dihydropyrimidinones [32]. The Biginelli reaction of acetoacetate, aromatic aldehyde and urea (thiourea) using trypsin from porcine pancreas as the catalyst has also been reported [33] (Scheme 4).

**Scheme 5:**
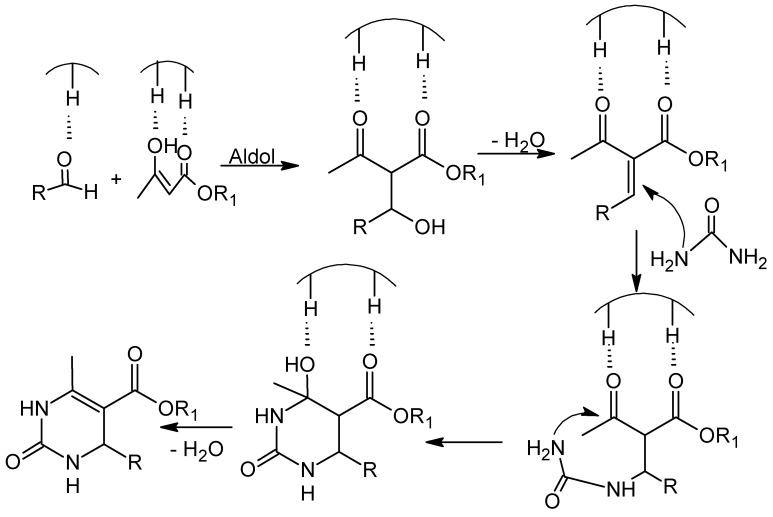
Mechanism of the enzyme-catalyzed Biginelli reaction.

Based on the results obtained, a general mechanism is proposed that starts with the activation of the carbonyl groups of the aceto ester and the aromatic aldehyde in the active site of the enzyme, followed by the aldol condensation with the aromatic aldehyde to generate the corresponding unsaturated β-ketoester. The latter undergoes a Michael addition of urea, followed by cyclization and dehydration to afford the product (Scheme 5).

Compared to some traditional methods [34,35,36], the above enzyme-catalyzed synthesis of 1,4-dihydropyridine (DHP) derivatives appears superior not only because the procedures are milder, simple in their operation and more environmentally friendly but, also, because they allow access to a wider variety of heterocycles in this category.

**Scheme 6:**
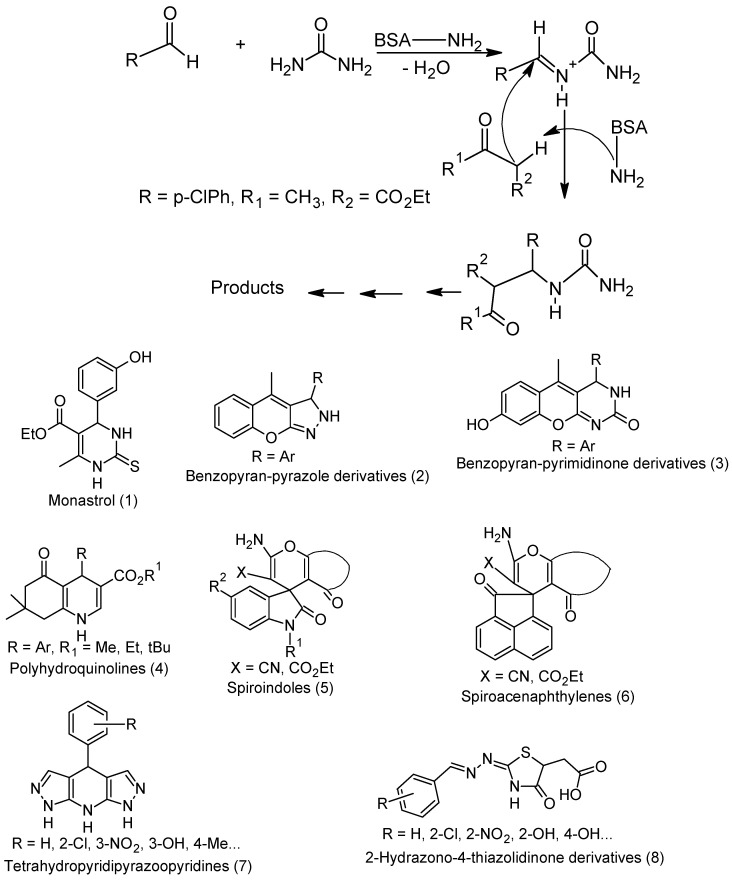
Mechanism of the bovine serum albumin (BSA)-catalyzed Biginelli reaction and some selected products accessible by this method.

The promiscuous activity of bovine serum albumin (BSA) was further demonstrated in the synthesis of DHPM and the corresponding thione derivatives by Sharma et al. [37], wherein a -gram scale synthesis of monastrol (1) (see Scheme 6), a potent mitotic kinesin Eg5 inhibitor, was obtained in 67% yield. The plausible mechanism suggested is depicted in Scheme 6 and is somewhat different from the one described in Scheme 5. In this case, the side chain amino acids of the enzyme play the role of the catalytic base and extract a proton from the active methylene compound to form a nucleophile, which reacts with an iminium ion arising from the reaction between the aldehyde and urea. Cyclization of this intermediate followed by dehydration, as described in Scheme 5, leads to the formation of the products.

**Scheme 7:**
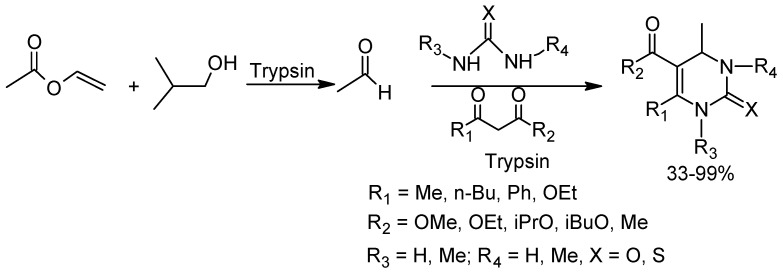
Enzyme in-situ generation of acetaldehyde in the Biginelli reaction.

The aldehyde components mostly used as partners in this MCR are aromatic aldehydes instead of acetaldehyde, because the latter is generally difficult to handle directly given its low boiling point, ease of polymerization and oxidation. In order to circumvent this drawback and expand the scope of the reaction, Wang and his group developed the first enzyme-catalyzed Biginelli reaction using acetaldehyde generated in situ by trypsin-catalyzed transesterification from vinyl acetate, combining two catalytic activities of one enzyme in a one-pot strategy for a two-step cascade reaction [38] (Scheme 7).

Urea and *N*-methylurea, as well as the corresponding thiourea derivatives, were tolerated in the reaction, affording a wide variety of heterocyclic compounds in high yields of up to 99%.

The biocatalytic preparation of a series of novel dihydropyrimidin-2(1*H*)-ones was achieved by the proper combination of *Rhizopus oryzae* lipase in a deep eutectic solvent consisting of a mixture of choline chloride and urea [16,39], giving rise to the expected products in good yields. This lipase in the deep eutectic medium gave high isolated yields in a short period of time. In addition, both the solvent and catalyst could be recycled up to four times without any major loss of activity, thus making the process both economical and environmentally friendly.

The versatility of the Biginelli reaction to rapidly generate biologically active heterocycles has been recently demonstrated in the synthesis of the benzopyran-connected pyrimidine (3) and benzopyran-connected pyrazole (2) derivatives via the reaction using Cu(II)-tyrosinase as a green catalyst [40] (see Scheme 6). The authors noted that literature observations showed that these coumarin derivatives are generally prepared by conventional methods using an acid catalyst while affording low yields and long reaction times. This procedure is, therefore, ecofriendly and gives high yields of 86–98%. The enzyme quickly catalyzed the cyclization reaction to afford compounds whose biotests exhibited larvicidal and antifeedant activities. In a related study, a series of pyrimidine-thione derivatives with potential mosquito larvicidal activity were prepared via a Cu(II)-tyrosinase catalyzed Biginelli reaction in up to 90% yield [41].

**Scheme 8:**
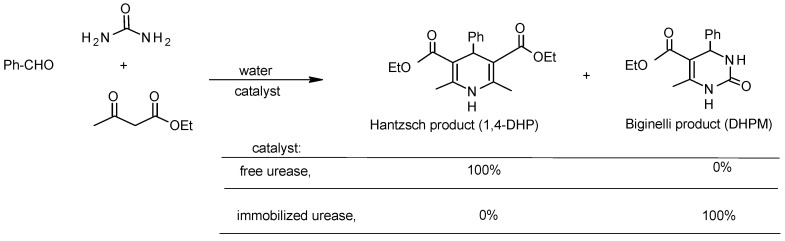
Catalytic effect of free and immobilized urease, leading to Biginelli and Hantzsch products. DHP: 1,4-dihydropyridine and DHPM: 3,4-dihydropyrimidin-2(1*H*)-one.

When urea or, indeed, a bifunctional nucleophile such as 5-aminopyrazole is used as the nitrogen source in a Biginelli reaction [42], Hantzsch products can potentially result under appropriate reaction conditions, such as the use of urease as a biocatalyst, and divergence between the two reactions is possible, leading to potential mixtures of DHMP and 1,4-dihydropyridine (DHP) derivatives. This competition between the two reactions was studied by Tamaddon et al. using urease immobilized on magnetic micro/nanocellulose dialdehydes. The immobilized urease catalyzed the Biginelli reaction with 100% selectivity in 68–92% yield, while the nonimmobilized enzyme, known to catalyze the hydrolysis of urea to ammonia, exclusively shifted the reaction to the Hantzsch product [43,44] (Scheme 8).

The main novelty in the above procedure is the immobilization of the enzyme on cellulose, a cheap, natural, biocompatible biopolymer that can easily be recycled. In addition, the enzyme denaturation due to its sensitivity and instability can be avoided thanks to the mild conditions during immobilization.

In another process, the synthesis of DHMP was catalyzed by a combination of the biocatalyst α-chymotrypsin in microwave irradiation at 55 °C in a circulating reaction system [45], demonstrating the synergistic effect of the microwave and the enzyme, thus greatly reducing the reaction time and increasing the yield.

## 4. The Hantzsch Reaction

**Scheme 9:**
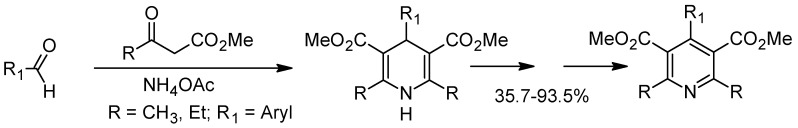
General Hantzsch pyridine synthesis.

The Hantzsch reaction, first reported in 1882, is a well-known protocol for the synthesis of 1,4-dihydropyridines (DHPs) from ethyl acetoacetate, an aldehyde and ammonia. It has undergone many modifications and improvements and is now the method of choice for fast access to DHP derivatives, which are useful for various bioactivities such as calcium channel blockers and antihypertensive agents [10,46] but, also, as scaffolds in medicinal chemistry [14]. Under oxidative conditions, these so-called Hantzsch esters can be transformed into the corresponding pyridines, a strategy used to prepare single regio-isomers of poly-substituted pyridines [47,48,49] (Scheme 9).

A novel and efficient synthesis of Hantzsch DHPs in the presence of fermenting baker’s yeast appeared in the literature in 2005. The acetaldehyde involved in the reaction resulted from an in-situ carbohydrate fermentation [50]. The same strategy was later used by Kumar and Maurya [32] to expand the scope of the reaction to more aldehydes and β-ketoesters, allowing for access to various polyhydroquinoline derivatives 4 (see Scheme 6) via an unsymmetrical Hantzsch reaction.

The source of ammonia in the reaction can be ammonium acetate, liquid ammonia, magnesium nitride [51] or urea. The unprecedented *Candida antarctica* lipase B-catalyzed (CAL-B) Hantzsch reaction using acetamide as the ammonia source was developed by Lin et al. [52], giving access to an array of 1,4-DHPs in excellent yields of up to 92%. On the other hand, when urea was used as the nitrogen source, the reaction was successfully catalyzed by urease leading selectively under appropriate reaction conditions to either Biginelli or Hantzsch products, as indicated earlier in Scheme 8.

**Scheme 10:**
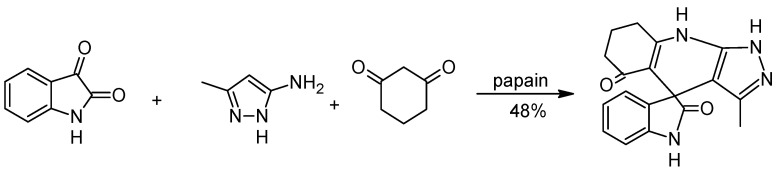
Papain-catalyzed Hantzsch synthesis of spiropyrazolo[3,4-b]pyridine.

Similarly, replacing the aldehyde by isatin, a one-pot papain-catalyzed synthesis of a series of spiropyrazolo[3,4-b]pyridines, starting from cyclic-1,3-diketones and 3-methyl-5-aminopyrazole, was achieved, affording highly substituted derivatives in moderate yields [53] (Scheme 10).

A related reaction with isatin and acenaphthenequinone was bio-catalyzed by swim bladders obtained from Caspian Sea fish (isinglass), leading to the domino synthesis of spiro-oxindoles and spiroacenaphthylenes 6 in water (Scheme 6) [54]. This is the first time this nontoxic, biocompatible and reusable biocatalyst, which required no special handling precautions, was used. Moreover, the present protocol is superior to the previous ones in terms of reaction time, yields and general reaction conditions.

**Scheme 11:**
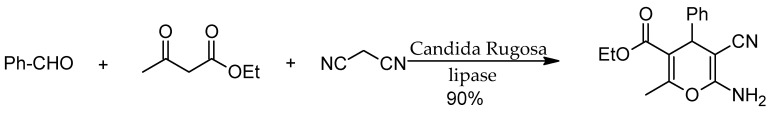
Lipase from *Candida rugosa* (CRL)-catalyzed Hantzsch-type synthesis of 2-amino-4*H*-pyrans.

Several enzyme-catalyzed three-component “Hantzsch-type” reactions where the amine partner is replaced by malonitrile, giving rise to several pyran derivatives, have appeared as an alternative access to this important class of compounds under mild, efficient and environment-friendly conditions [55,56,57] (Scheme 11).

Recently, a Hantzsch-type four-component synthesis of tetrahydrodipyrazolo-pyridines 7 (Scheme 6) from ethyl acetoacetate, an aldehyde, hydrazine and ammonium acetate was introduced. This ecofriendly protocol uses ovalbumin obtained from egg whites as an environmentally benign biocatalyst [58].

The stereochemistry of most of the above Hantzsch reactions was not reported, implying that the products obtained may have been racemic mixtures. Yet, enzymes are well-known to exhibit excellent control of the reaction stereoselectivity [59], resulting in a predefined absolute configuration of a specific stereocenter. Since different enantiomers of many DHP calcium blockers have different pharmacokinetic properties [60,61,62], the control of product enantioselectivity in Hantzsch synthesis is yet to be developed.

## 5. The Strecker Reaction

**Scheme 12:**
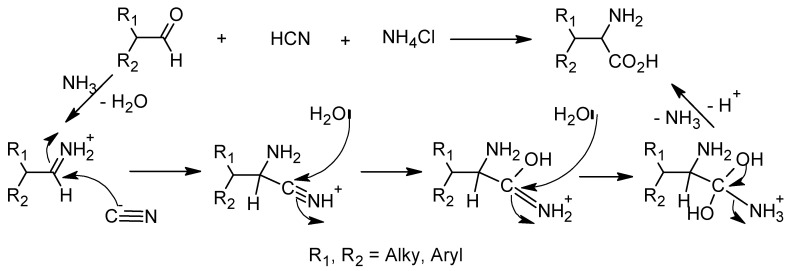
General Strecker reaction.

The discovery of the Strecker reaction (Scheme 12) made it possible to readily synthesize the building blocks that make up our proteins and enzymes. The first and interesting approach towards a biocatalytic asymmetric Strecker reaction was reported by Ramström and Vongvilai, in which they combined transamination with imine-cyanation under thermodynamic control and, subsequently, coupled in a one-pot process with a lipase-catalyzed transacylation under kinetic control [63].

Kawara et al. reported the chemo–enzymatic catalyzed synthesis of (*R*)-phenylglycine derivatives in high yields (79.5–97.8%) and high selectivity (86.5–98.7% ee) from benzaldehyde, ammonium buffer and potassium cyanide [64]. They used nitrilase AY487533 to catalyze the reaction, which led first to the formation of the corresponding racemic α-amino nitriles, whose R-enantiomer preferentially hydrolyzed to the acid than the S-enantiomer, which, under the equilibrium conditions of the Strecker reaction, reverts back to the starting materials. The forward reaction then regenerates the enzyme substrate, and the process is repeated until complete consumption of the starting materials. Low concentrations of the reagents and strict temperature and pH control are key to the success of the reaction.

The rational design of an enzyme-catalyzed multicomponent Strecker reaction can lead to the asymmetric synthesis of α-amino acids [65,66,67], thus avoiding the classical multistep procedures consisting, among others, of the resolution of racemic mixtures. Unfortunately, the above two examples of bio-catalyzed Strecker reactions appear to be the only ones described to date and, therefore, clearly need further developments.

## 6. The Mannich Reaction

**Scheme 13:**
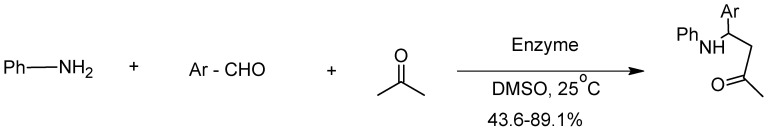
Lipase-catalyzed Mannich reaction.

The Mannich reaction is a powerful tool in organic synthesis that consists of an attack of a primary or secondary amino nitrogen on the carbonyl carbon of formaldehyde, resulting in an intermediate iminium ion (pH ~ 4 to 5), which then subsequently reacts with an enolisable carbonyl compound to generate β-aminoketone derivatives, also known as Mannich bases [68,69]. To exploit this powerful synthetic tool and access more complex molecular structures, formaldehyde is generally replaced by substituted benzaldehydes while anilines replace the amines. The first lipase-catalyzed direct, three-component Mannich reaction from a ketone or aldehyde and an amine to form β-aminoketone compounds was reported in 2009 by Yu et al. [70] (Scheme 13).

A series of substrates were explored in this reaction, resulting in moderate-to-good yields using various enzymes in water. However, lipase from *Mucor miehei* (MML) gave the best results, while lipase CAL-B showed moderate catalytic activity. Nevertheless, no selectivity was reported in all cases studied [71,72,73]. It was only a few years later that Xue et al. reported what is believed to be the first truly enzyme-catalyzed, direct three-component asymmetric Mannich reaction using protease type XIV from *Streptomyces griseus* [74]. The reaction was carried out in acetonitrile, affording up to 92% yield, 88% ee and 92:8 diastereoselectivities (syn:anti). Controlled experiments confirmed the enzyme catalytic action, mainly directing the stereoselective pathway of the reaction [16]. Encouraged by these results, Guan et al. studied the activity of another enzyme, acylase I (aminoacylase) from *Aspergillus melleus*, which catalyzes the hydrolysis of l-acyl-amino acids to produce the corresponding l-amino acids [75]. This enzyme activity and stereoselectivity were improved by adjusting the solvent, pH, water content, temperature, molar ratio of substrates and enzyme loading. Enantioselectivities of up to 89% ee, diastereoselectivities of up to 90:10 diastereoisomeric ratio (syn/anti) and yields of up to 82% were achieved.

In their efforts to assess the generality of the promiscuous lipase-catalyzed Mannich reaction, He et al. [71] identified lipase from *Candida rugosa* (CRL) as the most active for the reaction of both aromatic aldehydes, aliphatic ketones and aniline. Under optimized conditions, the Mannich adducts could be isolated in 20–94% with moderate diastereoselectivity.

**Scheme 14:**
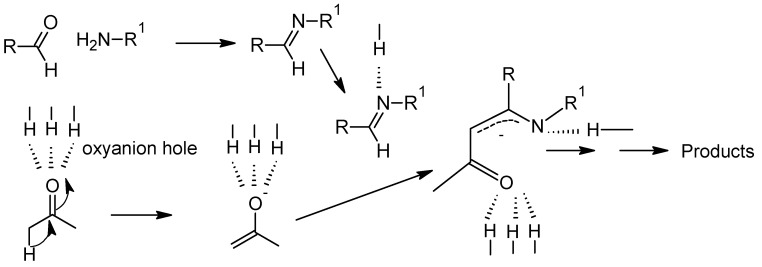
Mechanism of the Lipase-catalysed Mannich reaction.

In each case, the mechanism starts with the formation of the Schiff base from the reaction of the amine and aldehyde. At the same time, the enolate anion is stabilized by the oxyanion hole, followed by the formation of the iminium ion in the acidic active site of the enzyme. The enolate anion then attacks the iminium ion, forming a new carbon-carbon bond. H-atom transfer to the O-atom from acetone takes place in a concerted process. Finally, the Mannich adduct is released from the oxyanion hole (Scheme 14).

**Scheme 15:**
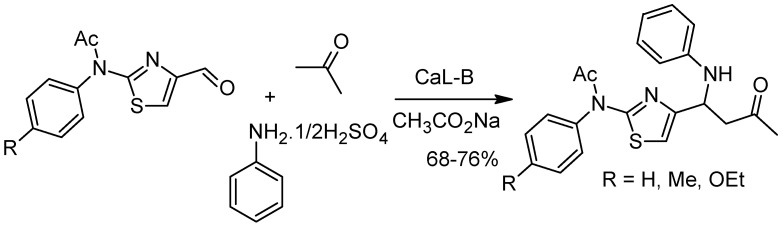
Synthesis of 2-phenylthiazole Mannich bases.

The catalytic activity of lipase from *Candida antarctica* lipase B (CAL-B) in Mannich reactions was studied by the group of Zaharia, who showed that this enzyme was very effective in the synthesis of new Mannich bases derived from 2-phenylthiazole and 2-phenylaminothiazole (Scheme 15). While the reaction worked well with acetone as the nucleophile component, affording the target compounds with good yields in mild and ecofriendly reaction conditions, this enzyme proved inactive with other ketones [76,77].

**Scheme 16:**
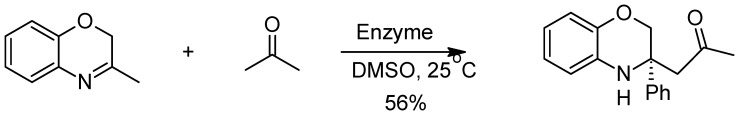
Lipase-catalyzed Mannich reaction of 3-phenyl-2*H*-1,4-benzoxazine.

Most reported bio-catalyzed Mannich reactions use the same type of starting materials: ketones, aryl amines and aromatic aldehydes. However, it can sometimes be necessary to preform the intermediate ketamine in a separate step and complete the Mannich reaction with the relevant ketone in the presence of the biocatalyst. This approach was used by the group of Wu [78], who studied the reaction of 3-phenyl-2*H*-1,4-benzoxazine with acetone in the presence of the cheap and readily available wheat germ lipase (WGL). Under optimized conditions, they obtained the corresponding Mannich adducts in moderate yields (56%) with a good enantioselectivity (87% ee) (see Scheme 16).

## 7. The Ugi Reaction

In its standard form, the Ugi reaction is a one-pot, four-component condensation reaction of a primary amine, a carbonyl compound, a carboxylic acid and an isocyanide to afford α-amino acid derivatives of a bis-amide product with the release of only one molecule of water as the only byproduct [79,80]. Due to its simplicity and applications in many fields, such as combinatorial, medicinal, peptide and peptidomimetics chemistry, this MCR rapidly developed to become one of the most versatile reactions in organic synthesis [81]. The acid component can be replaced by an acidic catalyst to form what is now known as a three-component Ugi reaction. Under these circumstances, enzymes are good candidates as possible catalysts.

**Scheme 17:**
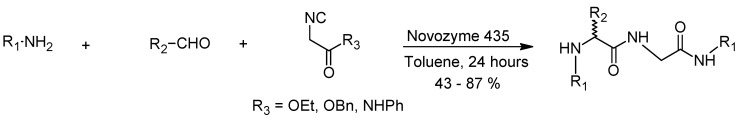
Enzyme-catalyzed Ugi reaction.

The first enzyme-catalyzed Ugi reaction, mediated by Novozym 435, was reported in 2013 by Kzossowski [80], allowing for the synthesis of a new class of dipeptides in up to 87% yield (Scheme 17).

Building on this experience, this same group replaced the aldehyde by a cyclic imine to develop an elegant synthesis of substituted pyrrolidines and piperidines catalyzed by Novozym 435, thereby expanding the activity promiscuity of this enzyme [82]. Later on, they further demonstrated that the enzyme catalyst selectivity was dependent on the chain length of the isocyanide ester, indicating a preference for short-chain isocyanoesters (C_1_ to C_4_) and switching the enzyme promiscuity between a three-component and a four-component MCR [83].

**Scheme 18:**
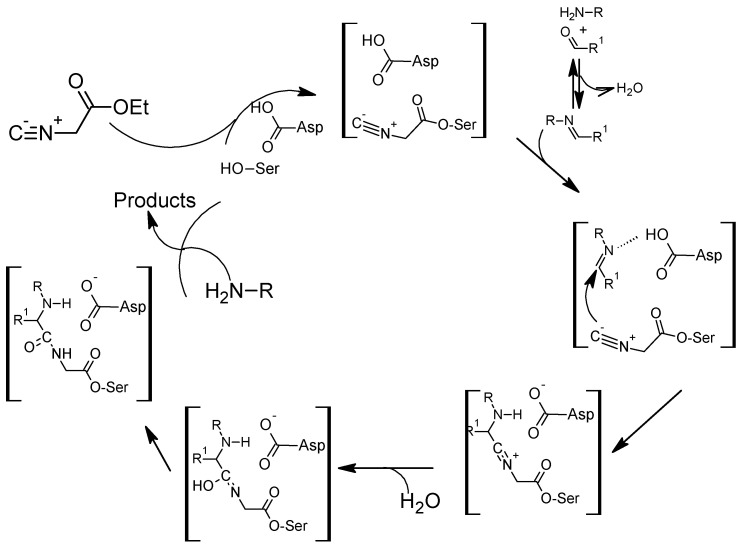
Mechanism of the enzyme-catalyzed Ugi reaction.

The mechanism of the reaction is depicted in Scheme 18. It is based on the traditional lipase-catalyzed ester hydrolysis, which involves the acylation by the ester of the isocyanide by a serine residue of the catalytic triad of the enzyme as the first step. Activation by acidic Asp residues of the Schiff base (formed from the reaction of the amine on the aldehyde) generates the corresponding iminium ion, which is attacked by the isocyanide nucleophile. The subsequent steps are similar to those of the classical Ugi reaction. The lack of stereoselectivity is attributed to reversibility of the reaction between the isocyanide and the iminium ion.

The above protocol allowed the first synthesis of dipeptide derivatives in Scheme 17 under mild, ecofriendly conditions in moderate-to-excellent yields.

## 8. Multicomponent No Named Reactions

There are a few multicomponent reactions catalyzed by enzymes but do not have special names. These are classified under this section according to the number of reactants involved in the reaction.

### 8.1. Three-Component Reactions

A series of 4H-pyran derivatives similar to those shown in Scheme 11 were synthesized by three-component reactions catalyzed by various enzymes [55,84,85,86].

In their search for potential new antidiabetic agents containing the hydrazono-4-thiazolidinone scaffold 8 (Scheme 6), Chavan et al. [87] designed an efficient and simple one-pot baker’s yeast catalyzed protocol for the synthesis of substituted derivatives of 2-hydrazono-4-thiazolidinone-5-acetic acids and the corresponding pyrazole analogs.

**Scheme 19:**
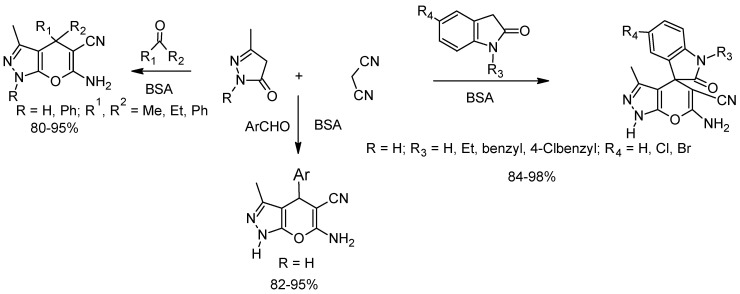
BSA-catalyzed synthesis of dihydropyrano[2,3-c]pyrazoles and spiro[indoline-3,4′-pyrano[2,3-c]pyrazoles].

The dihydropyrano[2,3-c]pyrazole skeleton (Scheme 19) can be accessed by a three-component reaction of aldehyde/ketone or isatin, malononitrile and 3-methyl-1*H*-pyrazol-5(4*H*)-one, catalyzed by BSA. The reaction is described to take place at ambient temperature in a mixture of ethanol and water to afford the products in 76–94% yield [88].

The same group later on developed a new synthesis of *ortho*-aminocarbonitriles from aromatic aldehydes, cyclohexanone and malononitrile using porcine pancreas lipase (PPL) as the catalyst [89]. In another report, a one-pot synthesis of spiropyrazolo[3,4-b]pyridine and spiro-oxindole scaffolds catalyzed, respectively, by papain and catalase, was carried out starting from isatin, 1,3-dicarbonyls and 3-methyl-5-amino pyrazole [90] (Scheme 6).

In addition to good-to-high yields, other advantages of this protocol include operational simplicity, simple filtration, no need for a chemical catalyst or activation and no column chromatographic purification and the biocatalyst tolerance to a wide range of substrates.

**Scheme 20:**
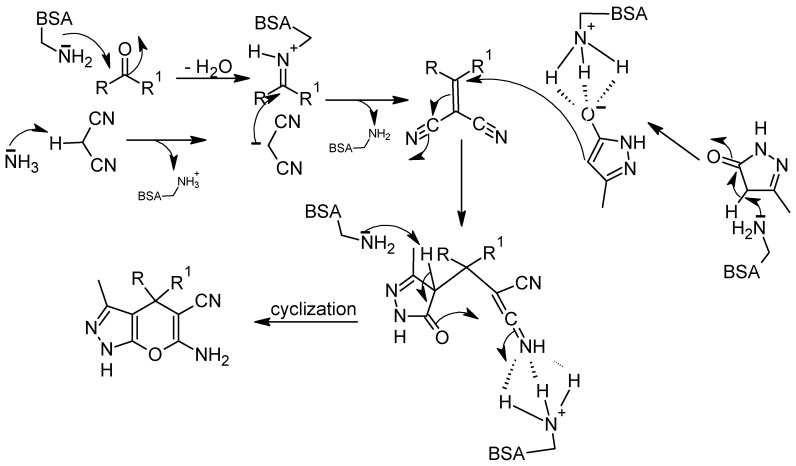
Mechanism of the BSA-catalyzed synthesis of dihydropyrano[2,3-c]pyrazoles and spiro[indoline-3,4′-pyrano[2,3-c]pyrazoles.

The proposed reaction mechanism begins with the normal formation of an iminium ion from the aldehyde and the basic lysine residues of the enzyme. At same time, the removal of the proton from malononitrile by the free basic amino group of BSA forms a carbanion that, subsequently, undergoes a Knoevenagel condensation with the iminium ion. The reaction intermediate condenses with the activated pyrazalone in a Michael addition reaction to form an adduct whose cyclization affords the product (Scheme 20).

### 8.2. Four-Component Reactions

**Scheme 21:**
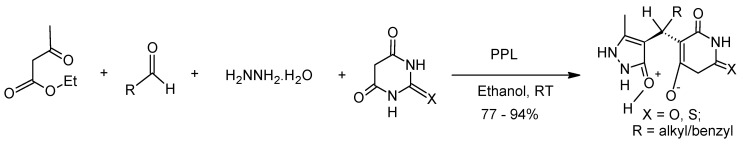
Porcine pancreas lipase (PPL)-catalyzed 4-component synthesis of pyrazalone derivatives.

Enzyme-catalyzed four-component reactions are not very common. The first reaction recorded in this category is the synthesis of dihydropyrano[2,3-c]pyrazoles [91] (see Scheme 19), mediated by *Aspergillus niger* lipase (ANL) from a mixture of ethyl acetoacetate, hydrazine hydrate, aldehyde/ketone and malononitrile. The reaction was carried out at room temperature and afforded excellent yields of the expected products. This report was followed by the porcine pancreas lipase (PPL) MCR developed by Bihani et al. [92] in what they termed the “pot, atom and step economic (PASE)” synthesis of 5-monosubstituted barbiturates bearing biologically active pyrazalone cores (Scheme 21), which were then screened against selected human pathogens with good pharmacological activity.

The mechanism of this reaction was not investigated, but the initial possible formation of a pyrazalone derivative followed by its condensation with the barbiturates was ruled out by the authors.

In another proof of its catalytic promiscuity, BSA was also shown to efficiently catalyze the one-pot four-component green synthesis of pyrano[2,3-c]pyrazoles (see Scheme 19). The catalyst could be recovered and reused up to five consecutive times with minimal loss of activity [93].

### 8.3. Five-Component Reactions

**Scheme 22:**
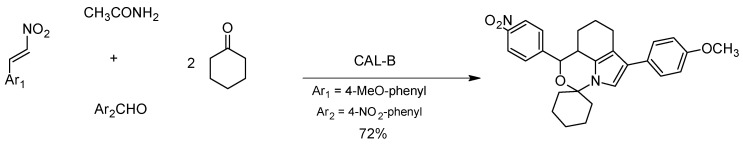
Enzyme-catalyzed 5-component synthesis of spiro-oxazino derivatives.

The group of Wu reported an aldol–Michael addition cascade reaction leading to the formation of six carbon–carbon/carbon–nitrogen bonds and two ring systems in a single operation [94] using *Candida antarctica* lipase B (CAL-B) as the biocatalyst (Scheme 22).

**Scheme 23:**
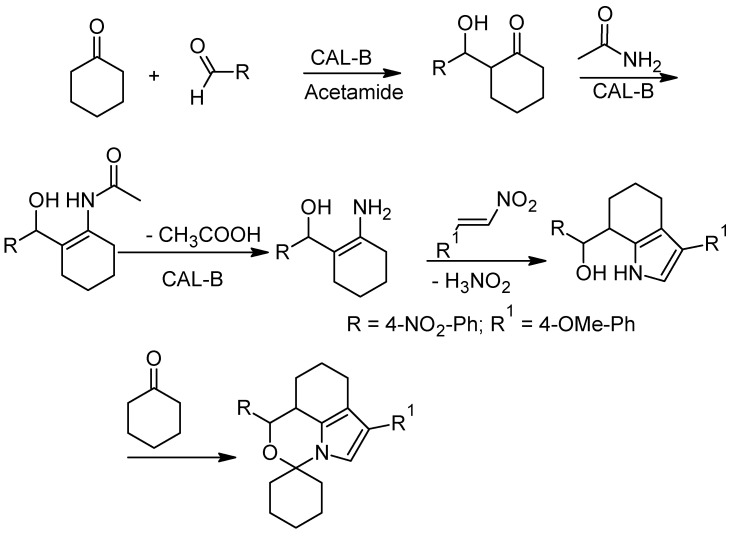
Mechanism of the enzyme-catalyzed 5-component synthesis of spiro-oxazino derivatives.

The reaction involved a lipase/acetamide-catalyzed Michael addition of ketones to nitroalkenes, affording spiro-oxazino derivatives. While the reaction worked well with *p*-nitrobenzaldehyde, low yields were obtained with other aldehydes. The proposed reaction mechanism involved an initial aldol condensation, followed by a Michael addition of the acetamide-activated aldol to the nitroalkene [95,96]. Cyclization of this adduct and the addition of a second molecule of the ketone leads to the final product as a racemic mixture (Scheme 23).

Based on these preliminary results, the authors were able to extend the scope of the reaction by using a preformed aldol compound. Under these new conditions, CAL-B catalyzed a two-step, one-pot multicomponent synthesis of spiro-oxazino derivatives, starting from readily available aldehydes, activated olefins, cyclohexanone and acetamide [97]. The first step consisted of the PPL-catalyzed asymmetric aldol reaction, followed after filtration of the PPL by the addition of CAL-B and all other substrates in the same pot, giving acceptable yields with diastereoselectivity and enantioselectivity of up to 93% ee. This approach opened up the door to many potential applications in the synthesis of complex organic molecules hardly accessible by other protocols.

## 9. Conclusions

This short review shows that bio-catalysis can be successfully applied to a wide variety of multicomponent reactions and constitute an ideal substitute to traditional catalysts in the viewpoint of efficiency, selectivity, yield and green sustainability. The stereochemical outcome of the Asinger, Mannich and Strecker reactions was successfully controlled, leading to the expected products with high ee and diastereoselectivity. However, only one example of enzyme-catalyzed reaction has been reported thus far for both the Asinger and Strecker reactions, despite the importance and versatility of the optically active intermediates derived from these reactions in the production of various pharmaceuticals and fine chemicals. These bioactive molecules obtained under a greener protocol offered by enzymatic catalysis is an attractive option for future scientific exploration.

The Biginelli reaction is, undoubtedly, the MCR most investigated using enzyme catalysis. While many Biginelli reaction products can be efficiently obtained using traditional non enzymatic procedures, such as under solvent-free and/or mechanochemical conditions, many of these methods use more expensive catalysts, sometimes times in stoichiometric amounts, require longer reaction times and generate waste materials. The advantage of bio-catalysis in this MCR is the accommodation of a broader substrate range under mild reaction conditions.

The Hantzsch reaction is closely related to the Biginelli reaction, and both lead to two scaffolds with several common structural features: 1,4-DHP (Hantzsch) and 3,4-DHMP (Biginelli), starting from the same reagents and with similar conditions. The use of enzymes as catalysts in these reactions opens new possibilities to access the two scaffolds under ecofriendly conditions. The formation of racemic products in the two MCRs and, indeed, in many other reactions needs further investigation to fully utilize the enzyme selectivity.

The Ugi reaction is among the most used diversity-oriented MCRs applicable in drug discovery, leading to the formation of peptide-like α-acylaminoamides containing one or more chiral centers in non-racemic forms, which are otherwise difficult to obtain by conventional methods. The benefits from the well-recognized facial discrimination brought about by enzyme catalysis is hereby highlighted.

Multicomponent reactions with four or more components are not very widespread, but progress continues to be made in the development of new MCRs, and they can benefit from the unique versatility of enzymes to accommodate various substrates.

While bio-catalysis in many classical reactions, such as the Michael reaction, aldol condensations, carbonyl reductions and ester hydrolysis, is well-established, its application in MCRs is still underdeveloped, and much work is required to harness its full potential.
